# MiR-195 affects cell migration and cell proliferation by down-regulating DIEXF in Hirschsprung’s Disease

**DOI:** 10.1186/1471-230X-14-123

**Published:** 2014-07-09

**Authors:** Hao Lei, Junwei Tang, Hongxing Li, Hongwei Zhang, Changgui Lu, Huan Chen, Wei Li, Yankai Xia, Weibing Tang

**Affiliations:** 1 State Key Laboratory of Reproductive Medicine, Institute of Toxicology, School of Public Health, Nanjing Medical University, Nanjing 211166, China; 2 Key Laboratory of Modern Toxicology (Nanjing Medical University), Ministry of Education, Nanjing, China; 3 Department of Pediatric Surgery, Nanjing Children’s Hospital Affiliated Nanjing Medical University, Nanjing 210008, China; 4 Department of Pediatric Surgery, Xuzhou Children’s Hospital Affiliated Xuzhou Medical University, Xuzhou 221002, China

**Keywords:** microRNA, Congenital diseases, Migration, Gastroenterology, HSCR

## Abstract

**Background:**

Hirschsprung’s disease (HSCR) is the most common congenital gut motility disorder. We aimed to investigate the roles of miR-195 in the pathogenesis of HSCR.

**Methods:**

In this study, we measured the expression levels of miRNA, mRNA, and protein in colon tissues from 78 patients with HSCR and 66 controls without HSCR. Transwell, Cell Counting Kit-8 (CCK-8) and flow cytometry assay were employed to detect the function role of miR-195 in vitro.

**Results:**

Our results showed that expression levels of miR-195 from patients with HSCR were significantly higher than control group; along with aberrant lower expression levels of digestive-organ expansion factor (DIEXF) were tested. Increased level of miR-195 could suppress the level of DIEXF in cell, which induced the impairment of cell migration and proliferation.

**Conclusions:**

Aberrant expression of miR-195 may involved in the pathogenesis of HSCR by down-regulated the level of DIEXF.

## Background

Hirschsprung’s disease (HSCR), also called aganglionosisis, is the commonest congenital gut motility disorder which is characterized by absence of the enteric nervous system in a variable portion of the distal gut during embryogenesis from 5 to 12 weeks [1]. It can lead to the clinical manifestation, which is often referred to as delay of meconium discharging and abdominal distension. The incidence of HSCR is different in different population, but is approximately 1:5000 in live births globally, while males are affected 4 times more often than females [2]. The current etiological studies of HSCR show that the manifestation of the disease is a complicated process involving both genetic and environmental factors. In particular, enteric neural crest cells (ENCCs) migration disorder could be important in the pathogenesis of HSCR. So far, more than 10 genes have been identified to be associated with the pathogenesis of HSCR, for example, RET, GDNF, SOX10 and so on [2-4].

MiRNAs are small, non-coding RNA molecules of 19–25 nucleotides which bind to 3′UTR of mRNAs and inhibit their expression either by interfering with translation or by destabilizing the target mRNAs [5]. To date, more than 800 miRNAs have been identified and many of them have been implicated in numerous biological processes, such as cell proliferation, migration, metabolism and apoptosis [6-9]. Recent studies have showed that miR-195 expression is decreased in many solid tumors, including gastric cancer, colorectal cancer and hepatocellular carcinoma [10-12]. Besides, miR-195 was reported to suppress osteosarcoma cell invasion and migration [13]. However, to our knowledge, few studies of miRNA in HSCR were reported. A better understanding of miRNAs during ENCCs development and effects of miRNAs in human HSCR generation is necessary.

## Methods

### Patients and samples

The study was approved by the institutional Review Board of Nanjing Medical University, and all subjects gave written informed consent. In the study, a total of 78 HSCR colon tissues were obtained from HSCR patients who underwent surgical treatment in Nanjing Children’s Hospital Affiliated to Nanjing Medical University from October 2009 to May 2012 (NJMU Birth Cohort). None of the patients had Syndromic HSCR. All the patients were diagnosed with barium enema anorectal manometry evaluation and rectal biopsies before surgical procedures. After surgery, pathological analysis was performed for definite diagnosis. We took specimens in the normal colon tissues of 66 patients without HSCR as a negative control. The samples were obtained from isolated patients received surgical treatment because of intussusceptions or incarcerated and strangulated inguinal hernia without the ischemia or necrosis parts. These were confirmed patients without HSCR or other congenital malformation. Finally, both the HSCR and control group samples were collected after obtaining informed consent from their guardians for the collection of the tissues. The tissues were immediately frozen and stored at -80°C after surgery.

### Quantitative RT-PCR

Quantitative RT-PCR was performed to determine the expression levels of miRNA and mRNA. Total RNA was obtained from tissues using TRIzol reagent as described by the manufacturer (Invitrogen Life Technologies Co, USA). TaqMan^®^ MicroRNA Assays (Applied Biosystems, CA, USA) was used as the probe for has-miR-195 and has-U6 which act as a normalized control. The detailed protocol was as described before [14]. Quantitative RT-PCR was performed using ABI Prism 7900HT (Applied Biosystems, CA, USA) according to the direction of the reagents. Forward (F) and reverse (R) primer sequences were as follows: DIEXF (F) 5′-ACAGCCAGTTCCTATCTGGTC-3′ and (R) 5′-GTAGAACAGGTCCCGGTAAGAA-3′; GAPDH (F) 5′-CACCGTCAAGGCTGAGAAC-3′ and (R) 5′-GGATCTCGCTCCTGGAAGATG-3′.

### Western blot

RIPA buffer which contains protease inhibitors (cOmplete, ULTRA, Mini, EDTA-free, EASYpack Roche, Germany) was applied to extract protein from gut tissues and BCA method was used to detected protein concentration. Equal amount of proteins (80 μg) were separated with 12.5% sodium dodecyl sulphate polyacrylamide gel electrophoresis (SDS-PAGE) and transferred to PVDF membrane (Roche Germany). Membrane was put into 5% skimmed milk for blocking and incubated with respective antibodies1. Primary polyclonal rabbit anti-DIEXF ab111508 was purchased from Abcam (Cambridge, MA, USA). The secondary antibodies were anti-rabbit HRP-linked which was purchased from Beyotime (Nantong, Jiangsu, China). The blots were developed using ECL reagent (Millpore, MASS, USA). GAPDH antibody was used to confirmed equal amount of protein loading in each lane. The integrated density of the band was quantified by ImageJ software.

### Dual-luciferase reporter assay

The full length 3′-UTR sequence of DIEXF were inserted into the KpnI and SacI sites of pGL3 promoter vector (Genscript, Nanjing, China). These constructs were named pGL3-DIEXF and pGL3-DIEXF-mut. Cells were plated onto 24-well plates and transfected with 100 ng of pGL3-DIEXF, pGL3-DIEXF-mut, 50nM miR-195 mimics and negative control, respectively, using lipofectamine 2000 (Invitrogen Corp, CA, USA). A Renilla luciferase vector pRL-SV40 (5 ng) was also co-transfected to normalize the differences in transfection efficiency. All experiments were repeated three times independently.

### Cell culture and reagents

Human SH-SY5Y cell was obtained from American Type Culture Collection (ATCC, Manassas VA, USA), which was cultured in complete growth medium DMEM (Hyclone, UT, USA), supplemented with 10% FBS, 100 U/ml penicillin, and 100 μg/ml streptomycin at 37°C, 5% CO_2_. Synthetic miRNA precursor molecules of *miR-195* and negative control (GenePharma, Shanghai, China) were used in transfection experiments.

### Cell transwell assays

For those cells treated with miRNA after transfection for 48 h, cells were seeded at 1 × 10^6^ cells/ml with serum-free medium, 100 μl cell suspension with serum-free medium was seeded to the upper chamber, cells were stained with crystal violet staining solution (Beyotime, Nantong, China) then counted and photographed under 40× magnification (five views per well). Migrated cells were counted using Image-pro Plus 6.0 while cell numbers of normal control group were normalized to 1. All experiments were performed in triplicate independently.

### Cell proliferation assays

CCK-8 assay (Beyotime, Nantong, China) was used to detect the cell proliferation. The TECAN infinite M200 Multimode microplate reader (Tecan, Mechelen, Belgium) was used to measure the absorbance at 450 nm. All experiments were performed in triplicate independently.

### Cell cycle and apoptosis analysis

Cells were transfected with *miR-195* mimics as well as negative controls for 48 h. All experiments were analyzed by BD Biasciences FACS Calibur Flow Cytometry (BD Biasciences, NJ, USA). All experiments were performed in triplicate independently.

### Statistical analysis

We used the method of 2-△Ct to analyze the results of RT-PCR in all the experiments performed in this study. Statistical analysis was performed using STATA 9.2, and presented with Graph PAD prism software. Experimental data of tissue samples are presented as box plot of the median and range of log-transformed relative expression level which was analyzed by Wilcoxon rank-sum (Mann–Whitney) test. The top and bottom of the box represent the seventy-fifth and twenty-fifth percentile. The whiskers indicate the 10th and 90th points. While the results obtained from experiment *in vitro* assays are presented as mean ± SEM from three separate experiments in triplicates per experiment, and the data was analyzed by double-sided Student’s t-test. Pearson correlation analysis was used to analyze the relationship of expression level of tissues between case and control group. Results were considered statistically significant at P < 0.05.

## Results

### Clinical information analysis

The clinical information, which including age, gender and weight were obtained among 78 HSCR patients and 66 normal controls without HSCR. The ages of HSCR patients and control groups were 3.4 ± 0.22 and 3.2 ± 0.27 months while the body weight were 5.7 ± 0.33 and 5.4 ± 0.28 kg, respectively. All these clinical information showed no statistical difference between HSCR patients and normal controls.

### Over-expression level of miR-195 in HSCR patients

**Figure 1:**
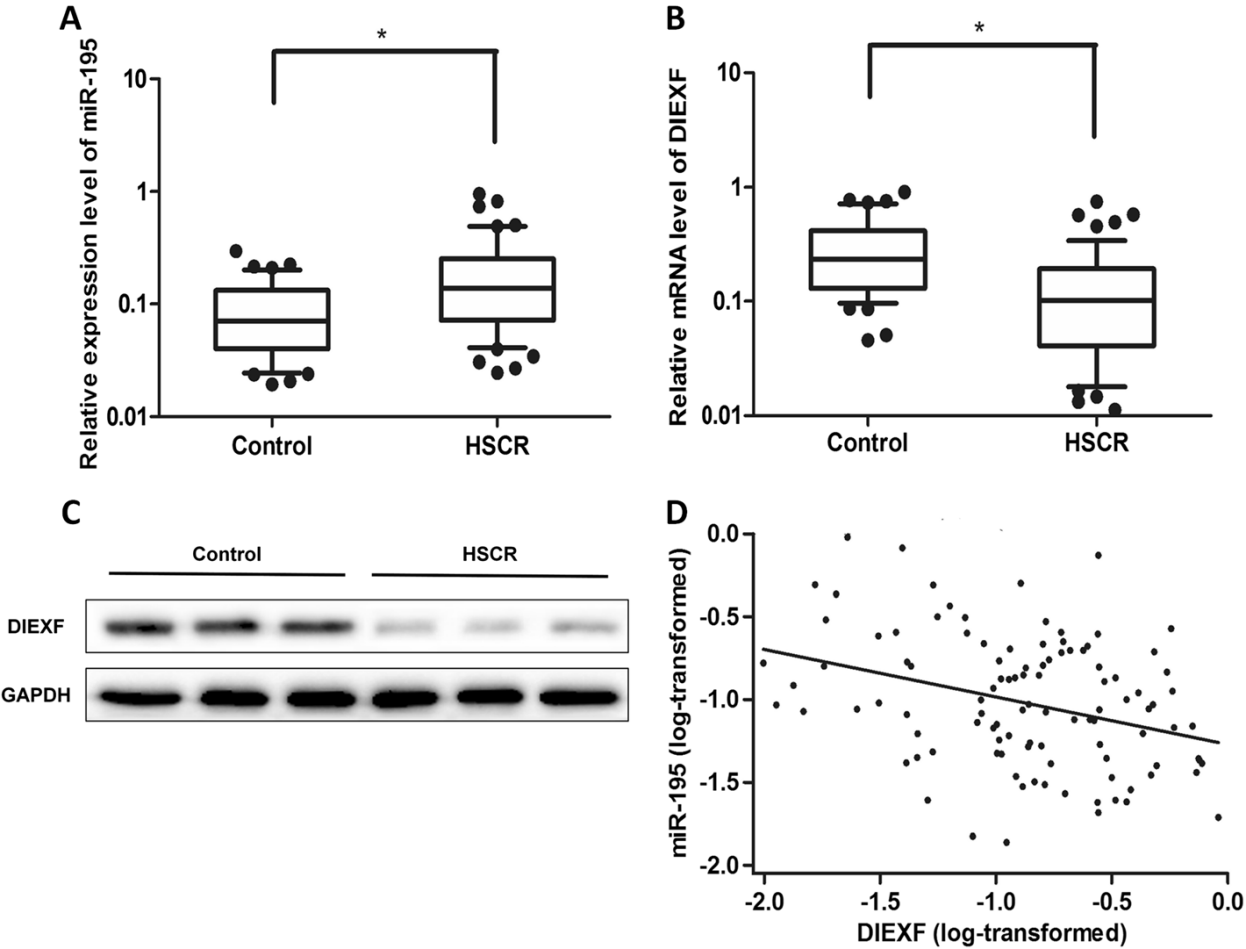
**Aberrant expression level of miR-195 and DIEXF.** All mRNA and miRNA expression levels were detected in HSCR tissues (n = 78) and control tissues (n = 66). **(A, B)**: Relative expression levels of miR-195 and DIEXF mRNA expression levels in HSCR tissues and control tissues. Data were presented as box plot of the median and range of log-transformed relative expression level. The top and bottom of the box represent the seventy-fifth and twenty-fifth percentile. The whiskers indicate the 10th and 90th points. **(C)**: Protein level confirmed the change of DIEXF. **(D)**: The correlations were analyzed between miR-195 expression and its corresponding DIEXF expression in HSCR tissues in bottom, P = 0.0001, R = -0.37. Data were analyzed using the Pearson correlation analysis with natural log transformed expression levels.

We analyzed miR-195 expression levels in HSCR patients and normal controls with Taqman quantitative real-time PCR methods. All relative expression levels of miR-195 were normalized to U6 expression levels. We obtained a result that miR-195 was markedly up-regulated in HSCR patients compared with normal controls, P = 0.0002, (Figure 1A). These findings directly suggest that miR-195 may play an important role in the appearance of HSCR.

### Expression levels of DIEXF were down-regulated in HSCR patients

To predict the functional target genes of miR-195, we employed bioinformatics approach. TargetScan (http://www.targetscan.org) database was employed to predict genes, which were potentially regulated by miR-195. Through this way, we obtained: DIEXF, ANO3, FGF2, SLC11A2 genes. To detect the expression levels of these genes, quantitative real-time PCR was employed. The mRNA expression levels of these genes were normalized to the level of GAPDH. DIEXF mRNA expression level was significantly down-regulated in HSCR patients compared with normal controls, P < 0.0001, (Figure 1B). However, the expression levels of ANO3, FGF2, and SLC11A2 showed no differences in HSCR patients and normal controls. We further tested the protein level of DIEXF, and we found that the protein level was consistent with its mRNA expression level (Figure 1C), The intergraded density of the bands was presented in Additional file 1: Figure S1A.

### Correlation between miR-195 and DIEXF expression levels

We analyzed the correlation between miR-195 and DIEXF relative expression levels in cases and controls. The analysis showed that a significant inverse correlation was observed between the levels of miR-195 and DIEXF, P = 0.0001, R = -0.37, which was supported by the Pearson correlation analysis (Figure 1D).

### MiR-195 mimics transfection decreased DIEXF expression

**Figure 2:**
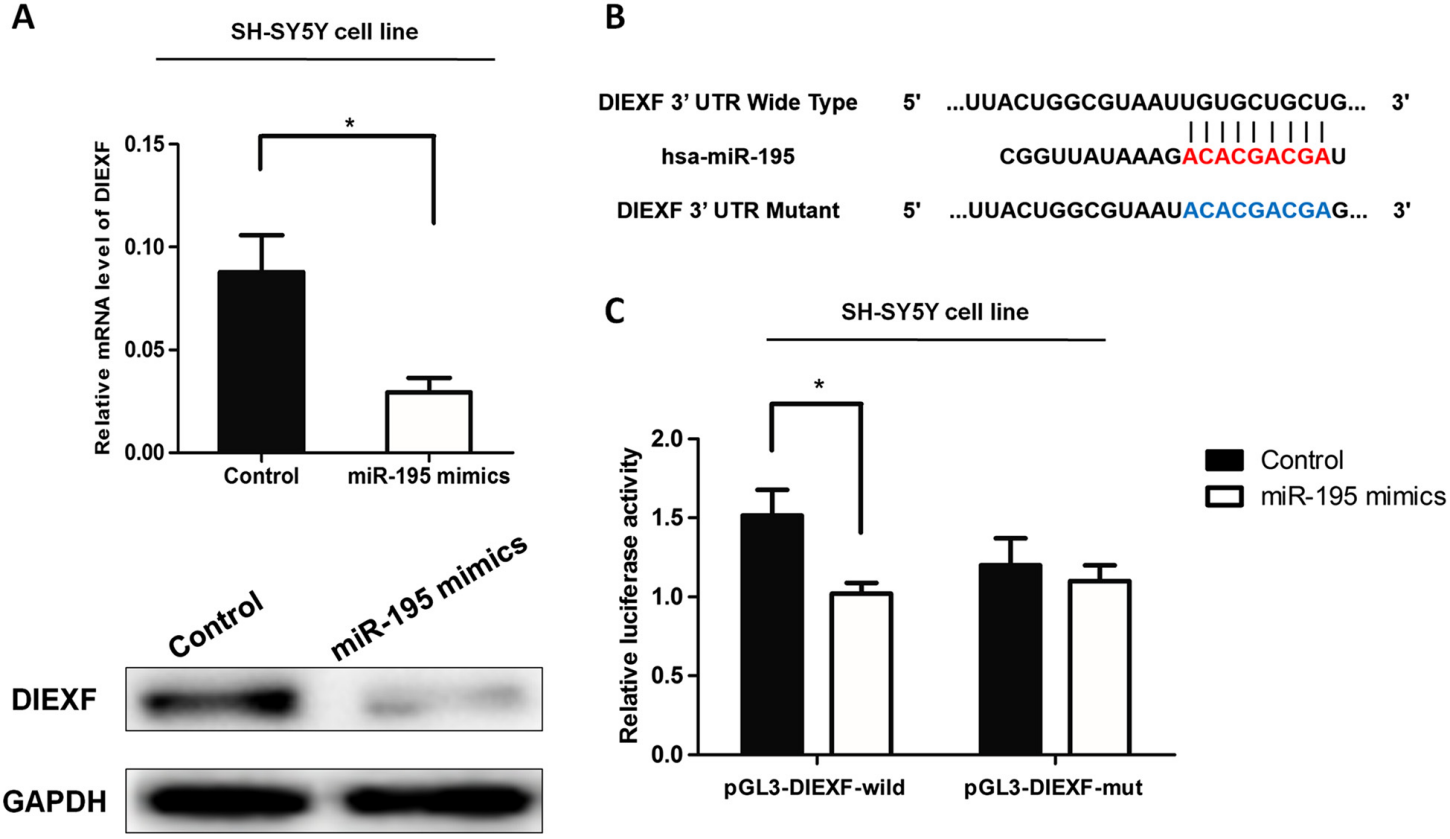
**Up-regulation of miR-195 reduced DIEXF mRNA and protein expression. (A)**: Cells were transfected with 50 nM miR-195 mimics for 48 h. qRT-PCR was performed to evaluate the mRNA level of DIEXF (top). Protein expression levels were also detected by western-blotting (bottom). **(B)**: Top: Sequence alignment of human miR-195 with 3′ UTR of DIEXF. Bottom: mutations in the 3′-UTR of DIEXF in order to create the mutant luciferase reporter construct. **(C)**: Cells were co-transfected with miR-195 mimics or miR-control, renilla luciferase vector pRL-SV40 and DIEXF 3′UTR luciferase reporter for 48 h. Both firefly and Renilla luciferase activities are measured in the same sample. Firefly luciferase signals were normalized with Renilla luciferase signals. All results were presented as mean ± SE. * indicates significant difference compared with that of control cells (P < 0.05).

We predicted that DIEXF might be the target gene of miR-195 by using bioinformatic methods. To validate the hypothesis, we transfected miR-195 mimics and normal control in the SH-SY5Y cell lines. The up-regulation of miR-195 was confirmed (Additional file 1: Figure S1B). DIEXF mRNA levels were evaluated after transfection for 48 hours. As expected, The results showed that the relative expression levels of DIEXF were down-regulated by miR-195 mimics transfection in SH-SY5Y cell lines (Figure 2A,B). Furthermore, to investigate whether the effect was due to direct binding of miR-195 to the 3′UTR regions of DIEXF, we applied miRNA luciferase reporter assay by constructing the wild type and mutant type luciferase reporter plasmids containing the binding region of the 3′UTR of DIEXF mRNA. We found that co-transfection of miR-195 mimic and pGL3-DIEXF 3′UTR reporter plasmids significantly decreased the luciferase activity in SH-SY5Y cell line as compared with the control (Figure 2C).

### Over-expression of miR-195 inhibited cell migration

**Figure 3:**
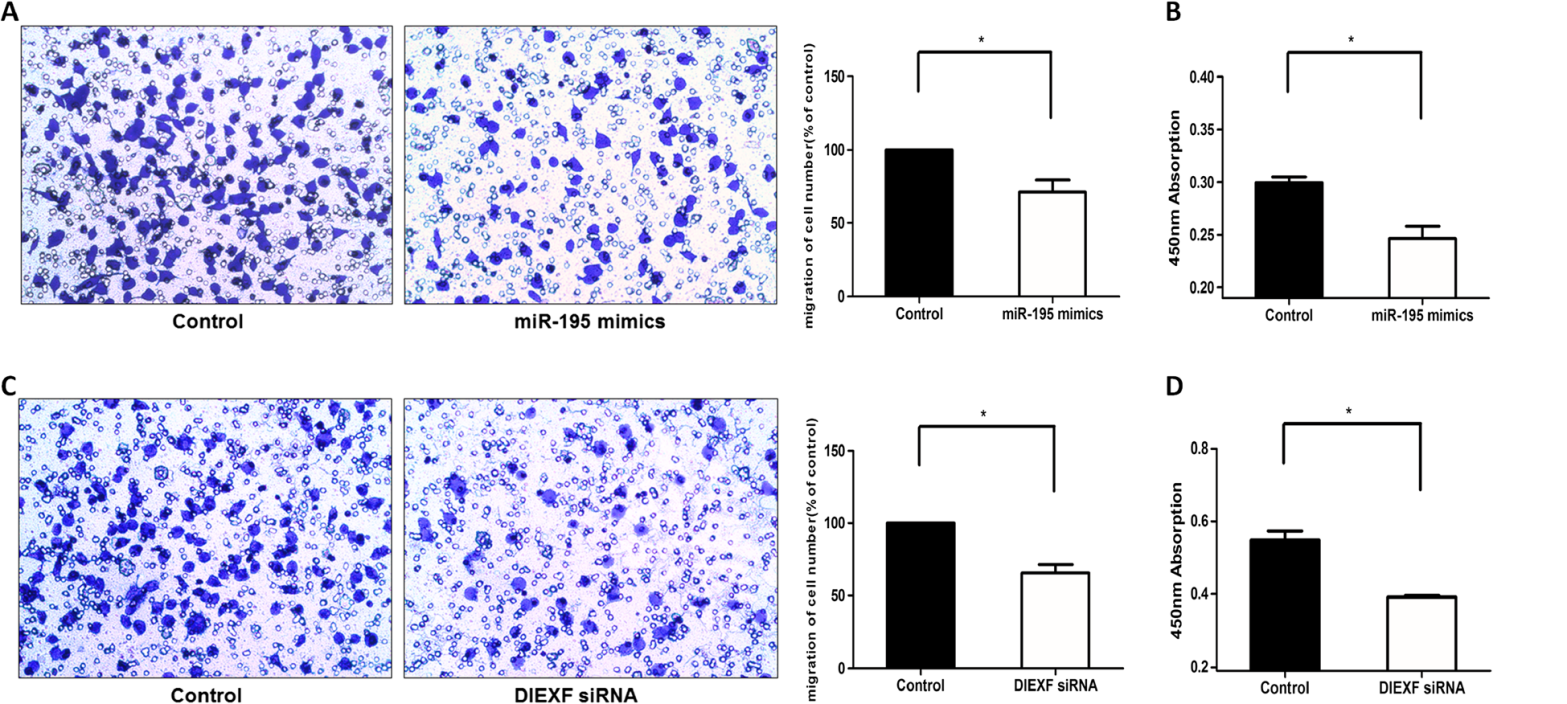
**Function of cytobiology alternation after miR-195 mimics (top) or DIEXF siRNA transfection (bottom) in SH-SY5Y cells. (A), (C)**: The representative images of invasive cells at the bottom of the membrane stained with crystal violet were visualized as shown (left). The quantifications of cell migration were presented as percentage migrated cell numbers (right). **(B), (D)**: Absorbance at 450 nm was presented with Mean ± SE. * indicates significant difference compared with control group P < 0.05.

To detect whether miR-195 plays an important role in the process of HSCR, we conducted cell migration assay. We transfected the SH-SY5Y cells with miR-195 mimic and normal control. The number of migrated cells of each image was numbered artificially. Migration of normal control group was normalized to 1. The results showed the number of migrated cell transfected with miR-195 was significantly fewer compared with the normal control (Figure 3A).

### Overexpression of miR-195 suppressed cell proliferation

By applying CCK-8 assay, we measured proliferation in the SH-SY5Y cell lines transfected with miR-195 mimic and normal control. We found that miR-195 mimic inhibited cell proliferation (Figure 3B) while did not affect the cell cycle or apoptosis (Additional file 1: Figure S1D).

### Decreased level of DIEXF suppressed cell migration and proliferation

To detect whether DIEXF plays an important role in the process of HSCR, we applied siRNA technology to knock down the expression level of DIEXF in human SH-SY5Y cells (Additional file 1: Figure S1C). We transfected the cells with DIXEF siRNA and negative control, the mRNA expression level of DIXEF was detected after transfecting for 48 hours. Our finds show that the number of migrated cell transfected with DIXEF siRNA was significantly fewer when compared with the normal control (Figure 3C). On the other hand, by using CCK-8 assay, we measured proliferation of cell lines transfected with DIXEF siRNA and negative control. As showed in Figure 3D, down-regulation of DIXEF inhibited cell proliferation.

## Discussion

MiRNA changes are common in several diseases and play an important role in disease development. In recent studies, miR-195 has been reported to be down-regulated in many solid tumors, including gastric cancer, colorectal cancer and hepatocellular carcinoma [15-17]. Also, it has been reported that high expression of miR-195 could suppress osteosarcoma cell invasion and migration [18]. So, we hypothesized that miR-195 functions as a tumor suppressor and inhibits cell migration and proliferation. In this study, we mainly detected the miR-195 expression levels and found that miR-195 was significantly up-regulated in HSCR patient colon tissues.

As we know, miRNAs are combined with 3′UTR of its target gene and take effect. We discovered DIEXF may be a target gene of miR-195 in TargetScan. DIEXF gene encodes a novel pan-endoderm-specific factor. Loss of function of DIEXF could lead to expansion growth arrest of digestive organs in zebrafish [19]. So, we monitored the expression of DIEXF and found an aberrant down-regulation of DIEXF in HSCR patient colon tissues.

We further hypothesized that high expression of miR-195 played an important way in pathogenesis of HSCR by targeting DIEXF. We design the experiment in vitro. When transfected miR-195 mimics in Human SH-SY5Y cell, we found that mRNA and protein expression levels of DIEXF were down-regulated. By using luciferase reporter, we found miR-195 directly regulated the DIEXF gene. What’s more, when miR-195 mimics were transfected in Human SH-SY5Y cell, cell migration and proliferation were suppressed. Besides, when decreasing the level of DIEXF, we found that cell migration and proliferation ability were inhibited. So, we got a conclusion that up-regulation of miR-195 and down-regulation of DIEXF might affect the ability of ENCCs migration and proliferation. Besides, Enteric neural crest cells (ENCCs) migration disorder sometimes was related with the abnormal extracellular matrix (ECM) [20]. Proteins of the matrix metalloproteinase (MMP) family are involved in the breakdown of extracellular matrix (ECM) in normal physiological processes including MMP2, 9. They encode two type of enzyme which degrades type IV collagen. Type IV collagen is one of ECMs and has been reported it is associated with the development of colonic aganglionosis. By transfecting miR-195 mimics and negative control in cells, we detected MMP2 and MMP9 expression. Results showed no effect on MMP2 and MMP9 of miR-195.

HSCR is characterized by the absence of the enteric nervous system in a variable portion of the distal gut. ENCCs migration disorder could be important in the pathogenesis of HSCR [21]. Any factor affecting proliferation, survival, migration, or differentiation of ENCCs might result in aganglionosis. In our previous studies on HSCR, we mainly focused on the genes in HSCR [14,22,23]. This study indicates that over-expression of miR-195 inhibits cell migration and proliferation as well as DIEXF did in human SH-SY5Y cell line. Moreover, the negative correlation between the expression levels of DIEXF and miR-195 and luciferase reporter indicates that DIEXF expression level is negatively regulated by miR-195.

## Conclusion

So, based on our present study, we can propose that miR-195-DIEXF pathway may play an important role in HSCR process by acting on ENCCs migration and proliferation. When miR-195 is up-regulated, the DIEXF gene will be down-regulated. Both of the two factors will affect the ENCCs migration and proliferation and lead to the arrest of ENCCs migration to the distal intestinal tract. And then, HSCR occurs.

However, miR-195 couldn’t be the only one miRNA which can lead to HSCR in the disease process. Further studies of the other miRNAs are needed. What’s more, the reason why miR-195 is up-regulated is still unknown. In future, more work need to be done and we hope our study can contribute to finding the pathogenesis of HSCR.

## Abbreviations

HSCR: Hirschsprung’s disease; ENCCs: enteric neural crest cells; 3′-UTR: 3′-untranslated region; DMEM: Dubelcco modified Eagle medium; FBS: fetal bovine serum; CCK-8: cell counting kit-8.

## Competing interests

The authors declare that they have no competing interests.

## Authors’ contributions

Conceived and designed the experiments: WT; Performed the experiments: HL; JT; HL; Analyzed the data: HZ; Wrote the paper: CL; HC; WL; YX. All authors discussed the results and commented on the manuscript.

## Pre-publication history

The pre-publication history for this paper can be accessed here:

http://www.biomedcentral.com/1471-230X/14/123/prepub

## Supplementary Material

Additional file 1: Figure S1 Additional information for the experiment in vitro. **(A)**: The intergrated density of DIEXF protein level in HSCR patient tissues and controls. **(B)**: The relative miR-195 expression level of SH-SY5Y cell line transfected with miR-195 mimics. **(C)**: Three site of DIEXF siRNA and the relative DIEXF expression level of SH-SY5Y cell line with DIEXF siRNA transfection. **D**: Over-expression of miR-194 did not affect the cell apoptosis or cell cycle. **E**: Western blot was detected in the expression of MMP2, 9 in cells transfected with miR-195 mimics and control. No significant difference was obtained in the two group. Click here for file
