# Supplementary figures and images for: MiR-195 affects cell migration and cell proliferation by down-regulating DIEXF in Hirschsprung’s Disease
Source: BMC Gastroenterol. 2014 Jul 9;14:123. doi: 10.1186/1471-230X-14-123 (PMC4099404; doi:10.1186/1471-230X-14-123)

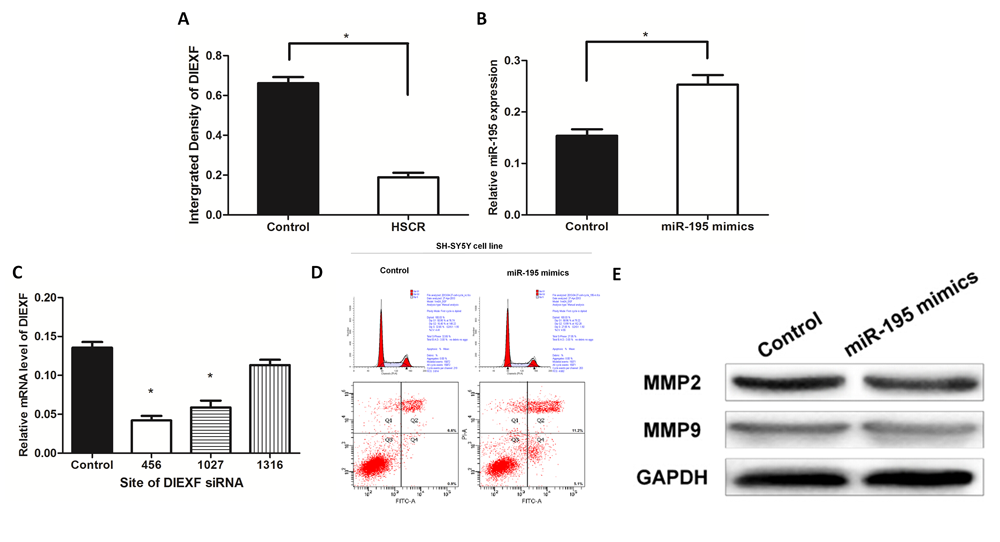

Supplement: Additional file 1: Figure S1 — Additional information for the experiment in vitro. (A): The intergrated density of DIEXF protein level in HSCR patient tissues and controls. (B): The relative miR-195 expression level of SH-SY5Y cell line transfected with miR-195 mimics. (C): Three site of DIEXF siRNA and the relative DIEXF expression level of SH-SY5Y cell line with DIEXF siRNA transfection. D: Over-expression of miR-194 did not affect the cell apoptosis or cell cycle. E: Western blot was detected in the expression of MMP2, 9 in cells transfected with miR-195 mimics and control. No significant difference was obtained in the two group. [file 1471-230X-14-123-S1.tiff]
